# Challenges and Opportunities of Intraoperative 3D Ultrasound With Neuronavigation in Relation to Intraoperative MRI

**DOI:** 10.3389/fonc.2021.656519

**Published:** 2021-05-03

**Authors:** Dhiego Chaves De Almeida Bastos, Parikshit Juvekar, Yanmei Tie, Nick Jowkar, Steve Pieper, Willam M. Wells, Wenya Linda Bi, Alexandra Golby, Sarah Frisken, Tina Kapur

**Affiliations:** Department of Neurosurgery, Brigham and Womens Hospital, Harvard Medical School, Boston, MA, United States

**Keywords:** ultrasound, 3D, neurosurgery, iMRI = intraoperative MRI, tumor

## Abstract

**Introduction:**

Neuronavigation greatly improves the surgeons ability to approach, assess and operate on brain tumors, but tends to lose its accuracy as the surgery progresses and substantial brain shift and deformation occurs. Intraoperative MRI (iMRI) can partially address this problem but is resource intensive and workflow disruptive. Intraoperative ultrasound (iUS) provides real-time information that can be used to update neuronavigation and provide real-time information regarding the resection progress. We describe the intraoperative use of 3D iUS in relation to iMRI, and discuss the challenges and opportunities in its use in neurosurgical practice.

**Methods:**

We performed a retrospective evaluation of patients who underwent image-guided brain tumor resection in which both 3D iUS and iMRI were used. The study was conducted between June 2020 and December 2020 when an extension of a commercially available navigation software was introduced in our practice enabling 3D iUS volumes to be reconstructed from tracked 2D iUS images. For each patient, three or more 3D iUS images were acquired during the procedure, and one iMRI was acquired towards the end. The iUS images included an extradural ultrasound sweep acquired before dural incision (iUS-1), a post-dural opening iUS (iUS-2), and a third iUS acquired immediately before the iMRI acquisition (iUS-3). iUS-1 and preoperative MRI were compared to evaluate the ability of iUS to visualize tumor boundaries and critical anatomic landmarks; iUS-3 and iMRI were compared to evaluate the ability of iUS for predicting residual tumor.

**Results:**

Twenty-three patients were included in this study. Fifteen patients had tumors located in eloquent or near eloquent brain regions, the majority of patients had low grade gliomas (11), gross total resection was achieved in 12 patients, postoperative temporary deficits were observed in five patients. In twenty-two iUS was able to define tumor location, tumor margins, and was able to indicate relevant landmarks for orientation and guidance. In sixteen cases, white matter fiber tracts computed from preoperative dMRI were overlaid on the iUS images. In nineteen patients, the EOR (GTR or STR) was predicted by iUS and confirmed by iMRI. The remaining four patients where iUS was not able to evaluate the presence or absence of residual tumor were recurrent cases with a previous surgical cavity that hindered good contact between the US probe and the brainsurface.

**Conclusion:**

This recent experience at our institution illustrates the practical benefits, challenges, and opportunities of 3D iUS in relation to iMRI.

## Introduction

Maximal safe resection of high-grade and low-grade gliomas has been established as an important prognostic factor with a strong correlation to survival (1,4). Image-guidance using computerized navigation on the basis of preoperative MRI is the current standard in the surgical management of brain tumors. Although neuronavigation greatly improves the surgeons ability to approach, assess and operate on brain tumors, navigation based on preoperative MRI loses its accuracy as the surgery progresses, owing to substantial brain shift and deformation (5,8). Intraoperative MRI (iMRI) partially addresses this inherent problem of neuronavigation based on preoperative imaging when used serially during the surgery to provide anatomical updates reflective of the changing tissue structure (9, 10).

The first iMRI system, developed in the 1990s by General Electric in collaboration with physicians at Brigham and Womens Hospital, was designed as a double donut configuration with the surgeon standing in the aperture between the two halves of the magnet. This provided the surgeon full access to the head without the need to move the patient in and out of the bore of the scanner (11). This system integrated navigation with continuous multi-oblique image plane acquisitions. This design mandated a low field strength magnet of 0.5T and was limited by the resultant poor image quality when compared to preoperative diagnostic MRI scanners of field strengths typically ranging from 1.5T to 3T. To improve image quality and resolution, higher field, closed configuration magnets are necessary, requiring either moving the patient deep into the bore of the magnet or moving the magnet to the patient on the operating room table. This requirement makes it impractical to acquire multiple images to update neuronavigation as tumor resection progresses and tissue deformation ensues, due to the time cost associated with each iMRI imaging session. Thus, the common practice in iMRI guided brain tumor surgery is to perform a single iMRI imaging session near the end of intended resection for identifying any residual tumor.

Intraoperative ultrasound (iUS) is a powerful alternative to iMRI for monitoring brain shift and updating neuronavigation during surgery. The main advantage of iUS is that it causes minimal disruption to the surgical workflow while providing real-time information to the surgeon and is much less expensive and resource intensive than iMRI (12). 2D iUS has the disadvantage of a steep learning curve and an increased cognitive burden necessitated to integrate the orientation of 2D images to the 3D anatomy of the surgical field. Thus, it can be difficult for surgeons to maximally benefit from 2D iUS in neurosurgical procedures. Integrating preoperative MRI with 3D iUS in neuronavigation helps resolve these orientation challenges of 2D iUS (13,15; Geirmund 16,18).

In this report, we present the challenges and opportunities in the use of 3D iUS during brain tumor resection in an advanced image-guided operating environment with multimodal preoperative MRI, neuronavigation and iMRI. We describe how we use preoperative MRI, iMRI, and 3D iUS intraoperatively and discuss the current and future impact of these imaging modalities on neurosurgical practice.

## Materials and Methods

We retrospectively evaluated patients who underwent image-guided brain tumor resection in the Advanced Multi-modal Image-Guided Operating (AMIGO) Suite (19, 20) at Brigham and Womens Hospital in Boston, USA, between June 2020 and November 2020, where both iUS and iMRI were employed to guide the resection. We selected cases representative of patients at higher risk of post-operative neurological complications due to the location of the tumor in or near the eloquent cortex or patients where intraoperative imaging was required to guide resection. The cases were individually analyzed and the roles of iUS and iMRI at multiple time points were compared. Clinical, demographic, histopathological and radiological information was manually collected from the patients electronic medical records. Tumors were classified according to the World Health Organization (WHO) 2016 Classification of Gliomas and cIMPACT-NOW updates 4, 6 and 7 (21,24). Additionally, tumors were classified into one of 3 categories based on proximity to functional cortex (non-eloquent [Grade I], near-eloquent [Grade II], and eloquent [Grade III]) (25). Extent of resection (EOR) was classified by a neuroradiologist as gross total resection (GTR) or subtotal resection (STR) based on a postoperative MRI performed within 48 hours after surgery. We also describe three cases in greater detail and note the nuances in our experience of using iUS in neurosurgical practice. The study was approved by the Mass General Brigham Institutional Review Board and written informed consent was obtained from all patients included in this study. The surgical procedures in this series were performed either by W.L.B or A.G, both neurosurgeons with extensive experience in image-guided neurosurgery using iUS and iMRI.

### Cohort Selection

The study was conducted between June 2020 and December 2020 when an extension of a commercially available navigation software was introduced in our practice enabling 3D iUS volumes to be reconstructed from tracked 2D iUS images. During this period, a total of 23 cases were performed in the AMIGO suite by the study surgeons. Preoperatively, MR images were acquired using a 3T MRI scanner (Magnetom Prisma/Skyra, Siemens Healthineers, Erlangen, Germany) and a 20-channel Siemens head-coil. Structural MR imaging included a 3D T1-weighted post-contrast sequence, a 2D T2-weighted sequence, a 3D MP2RAGE sequence, and a 3D T2-weighted FLAIR sequence. Patients with lesions in or near eloquent areas underwent blood oxygen level-dependent (BOLD) fMRI with a single-shot 2D echo-planar imaging (EPI) sequence with the appropriate tasks paradigms for language and motor mapping, according to our institutional protocol (26). Preoperative Diffusion MRI (dMRI) was acquired in all patients and Brainlab Fibertracking software module (Brainlab Elements, Munich, Germany) was used for tractography in patients who had lesions in close proximity to eloquent white matter tracts. Relevant fMRI activations were used as seed regions to generate fiber tracts, either individually or in combination, as deemed clinically appropriate. All MR images were imported into a neuronavigation system (Brainlab, Munich, Germany) for presurgical planning.

### 3D Intraoperative Ultrasound (iUS)

iUS was performed using a 2D neuro-cranial curvilinear transducer on a cart-based ultrasound system (N13C5, BK5000, Analogic Corporation, Peabody, MA, USA). This sterilizable transducer has a contact surface area of 29x10 mm and frequency range of 13-5 MHz. The ultrasound probe was tracked with the Brainlab Curve navigation system (Brainlab, Munich, Germany). Similar to optical tracking of other surgical instruments, a fiducial array was attached to the ultrasound transducer and tracked relative to a reference array attached to the head fixation device (HFD100, IMRIS, Minnetonka, MN, USA). This allowed the navigation software to localize the acquired 2D iUS slices and orient them at the transducer tip. During image acquisition, the imaging plane of the transducer was oriented as close as possible to one of the three cardinal axes of the head, determined by the size and shape of the craniotomy (e.g., when the largest dimension of the craniotomy was along the anteroposterior axis, the transducer was placed in a coronal or sagittal orientation depending on the surgeons view preference). The 2D probe was slowly swept across the craniotomy to acquire a sequence of tracked 2D images. A 3D iUS volume was created from the tracked images at a resolution of 0.2mm x 0.2mm x 0.2mm using an automated ultrasound reconstruction extension at the backend of the navigation software (Digital Ultrasound Integration, Brainlab, Munich, Germany). The 3D iUS volume was overlaid on preoperative imaging, providing insight into brain shift and possible registration errors. For each patient, three or more 3D iUS volumes were acquired during the procedure, and one iMRI was acquired after significant resection towards the end of the procedure. The iUS images included: an extradural image acquired immediately after the craniotomy and before dural incision (iUS-1); an intradural image acquired immediately after the dural opening (iUS-2); and a third image acquired immediately before the iMRI acquisition (iUS-3) (S. 27).

### Intraoperative MRI (iMRI)

iMRI was performed using a 3T wide-bore (70cm) MRI scanner (Magnetom Verio, Siemens Healthineers, Erlangen, Germany) after significant resection to evaluate the presence of any residual tumor. A temporary closure was performed prior to the iMRI acquisition. The total time required to obtain iMRI including preparation for scanning, MR safety check, image acquisition, and repreparing for surgery t was 1-1.5 hours per session. In some of the surgeries, clinical needs necessitated imaging sessions at 2 distinct surgical timepoints. The Brainlab registration module was used to automatically register iMRI to preoperative MRI (rigid registration). This allows us to compare iMRI in the same coordinate space as iUS.

### Comparison of iUS to Neuronavigation and iMRI

iUS-1 and iUS-2 were used to provide a real-time assessment of the lesion extension, location and local anatomy and to confirm navigation accuracy. iUS-3 was qualitatively compared to iMRI to evaluate how well the iUS could image residual tumor and predict the extent of the resection. The presence or absence of residual tumor on iUS-3, as per the attending neurosurgeon, was recorded prior to the acquisition of the iMRI. The assessment of the residual tumor in iMRI was also made by the attending neurosurgeon in consultation with a neuroradiologist.

## Results

We compared the clinical utility of iUS with preoperative MRI and iMRI in twenty-three patients (15 men, 8 women; age range 28-83 years) who underwent image-guided brain tumor resection in the AMIGO suite (**Table 1**). In seven patients, the tumors were located in eloquent brain regions, eight near-eloquent brain regions, and eight in a deep-seated non-eloquent brain region. Thirteen patients had newly diagnosed tumors, while ten patients underwent resection for recurrent tumors. Five patients had *IDH1-*mutant grade 3 gliomas, three patients had an integrated histological and molecular diagnosis of glioblastoma multiforme, ten had grade 2 gliomas, one had brain metastases from a non-small cell lung carcinoma, one had a diagnosis of radiation necrosis and one had a central nervous system lymphoma. GTR was achieved in twelve patients (in the other patients, subtotal resection was expected due to the unfavorable location of tumors in the eloquent cortex). Temporary postoperative deficits were observed in five patients, all of whom had tumors in eloquent or near-eloquent regions. **Table 2** summarizes tumor pathology along with intraoperative details of the tumor resection and the postoperative patient outcomes.

**Table 1:** Summary of patient demographics, and preoperative clinical and radiological aspects of brain tumors.

#	Sex (Age, years)	Tumor Location	Recurrent	Eloquence	Related White Matter Tracts	Contrast Enhancing
1	Male (61)	Right frontal	No	Near Eloquent	CST	No
2	Male (28)	Left medial temporal	No	Near Eloquent	IFOF, Arcuate, visual fibers	No
3	Female (53)	Left cingulate gyrus	No	Near Eloquent	CST, Frontal Aslant Tract	No
4	Male (58)	Left Insula	No	Eloquent	CST, Arcuate	No
5	Male (45)	Right temporal-Recurrent	Yes	Non-eloquent		Yes
6	Male (36)	Left insula	Yes	Eloquent	CST, Arcuate	No
7	Male (58)	Left frontal	No	Eloquent	CST	Yes
8	Female (34)	Right Insula-Recurrent	No	Eloquent	IFOF, Uncinate	No
9	Male (83)	Right frontal	No	Eloquent	CST	Yes
10	Male (52)	Left temporal	No	Near Eloquent	CST, Arcuate	Yes
11	Female (56)	Left frontal	Yes	Non-eloquent		Yes
12	Female (32)	Left temporal	Yes	Near Eloquent	Arcuate, IFOF	No
13	Female (49)	Left frontal	Yes	Non-eloquent		Yes
14	Male (45)	Right frontal	No	Near Eloquent	CST	No
15	Female (45)	Right parietal	Yes	Near Eloquent	CST	No
16	Male (42)	Left frontal	Yes	Non-eloquent		Yes
17	Female (23)	Left occipito-parietal	Yes	Non-eloquent		Yes
18	Male (48)	Right frontal	Yes	Non-eloquent		Yes
19	Male (52)	Right temporal	No	Non-eloquent	IFOF	No
20	Male (42)	Right precentral gyrus	No	Eloquent	CST	No
21	Female (23)	Right temporal	No	Non-eloquent		Yes
22	Male (69)	Left frontal	Yes	Eloquent	Arcuate, CST	No
23	Male (54)	Left frontal/insula	No	Near Eloquent	IFOF, Arcuate	No

Corticospinal Tract; Inferior fronto-occipital fascicle.

**Table 2:** Summary of Intraoperative and Postoperative Imaging Findings.

#	Lesion echogenicity	Predura US Anatomical Landmark	US1 confirms navigation MRI	Residual Tumor (US)	Residual Tumor (iMRI)	Extent of Resection	Postop Deficits	Tumor Pathology
1	Homogeneous hyperechoic	Sulci	Yes	No	No	GTR	None	GBM**
2	Heterogeneous hyperechoic	Tentorium, brainstem, cerebellum	Yes	Yes	Yes	STR	Temporary word findings and reading difficulty	Anaplastic Astrocytoma *IDH1*mutant, Grade 3
3	Homogenous hyperechoic	Falx, cingulate sulcus, lateral ventricles, callosum sulcus	Yes	Yes	Yes	STR	SMA* syndrome	GBM**
4	Homogeneous hyperechoic	Sylvian fissure	Yes	Yes	Yes	STR	Temporary aphasia	Diffuse Astrocytoma *IDH1*mutant, Grade 2
5	Heterogeneous hyperechoic	Lateral ventricle, tentorium	Yes	Yes	Yes	STR	None	Anaplastic Oligodendroglioma, Grade 3
6	Homogeneous hyperechoic	Sylvian fissure, ventricles	Yes	Yes	Yes	STR	None	Anaplastic Astrocytoma *IDH1*mutant, Grade 3
7	Hypoechoic cyst + hyperechogenic margins	Falx, ventricles	Yes	No	No	GTR	None	Metastasis (NSCLC)
8	Heterogeneous hyperechoic	Sylvian fissure, Falx	Yes	Yes	Yes	STR	None	Diffuse Astrocytoma *IDH1*mutant, Grade 2
9	Isoechoic with surrounding hyperechoic edema	Lateral ventricles, Falx	Yes	No	No	GTR	Temporary UE paresis	Lymphoma
10	Heterogeneous hyperechoic solid component & hypoechogenic cyst	Occipitotemporal sulcus	Yes	Yes	Yes	GTR	No	Oligodendroglioma, Grade 2
11	Heterogeneous hyperechoic	Orbital gyri	Yes	Yes	Yes	GTR	No	Radiation necrosis
12	Heterogeneous hyperechoic	Tentorium, brainstem, cerebellum	Yes	Yes	Yes	STR	No	Diffuse Astrocytoma *IDH1*mutant, Grade 2
13	Nonvisible (large surgical cavity)	Ventricles	No	Not visible (previous resection cavity)	Yes	GTR	No	Anaplastic Oligodendroglioma, Grade 3
14	Homogeneous hyperechoic	Sylvian fissure	Yes	Yes	Yes	STR	No	Oligodendroglioma, Grade 2
15	Heterogeneous hyperechoic (hard to visualize)	Lateral ventricles	Yes	Not visible (previous resection cavity)	No	GTR	No	Oligodendroglioma, Grade 2
16	Heterogeneous hyperechoic	Lateral ventricles	Yes	Yes	Yes	STR	No	Oligodendroglioma, Grade 2
17	Heterogeneous hyperechoic	Falx	Yes	Not visible (previous resection cavity)	No	GTR	No	Grade 2 low grade glioma with ependymoma differentiation, IDH1 wildtype
18	Homogeneous hyperechoic	Lateral ventricles	Yes	Not visible (blurry)	Yes	GTR	No	Anaplastic Astrocytoma *IDH1*mutant, Grade 3
19	Homogeneous hyperechoic	Sylvian fissure	Yes	Yes	Yes	GTR	No	Oligodendroglioma, Grade 2
20	Homogeneous hyperechoic	Sulci	Yes	Yes	Yes	GTR	No	Oligodendroglioma, Grade 2
21	Hypoechoic cyst + hyperechogenic margins	Sylvian fissure	Yes	No	No	GTR	No	Ganglioglioma, grade I
22	Heterogeneous hyperechoic	Lateral ventricles	Yes	Yes	Yes	STR	No	Anaplastic Oligodendroglioma, Grade 3
23	Heterogeneous hyperechoic	Lateral ventricles	Yes	Yes	Yes	STR	Status epilepticus	GBM

Gross total resection; Subtotal resection; Non small cell lung cancer; Upper extremity; *Supplementary motor area; **Glioblastoma multiforme.

In all cases, iUS images acquired at two time points were visually compared with either preoperative MRI or iMRI. In nineteen patients, the iUS and iMRI findings regarding the status of the resection (GTR or STR) were concordant. In all these patients, iUS was able to define tumor location, tumor margins and was able to indicate relevant landmarks for orientation and guidance. The remaining four patients where iUS was not able to evaluate the presence or absence of residual tumor were recurrent cases with a previous surgical cavity that hindered good contact between the US probe and the brain surface. In sixteen cases, white matter fiber tracts computed from preoperative dMRI were overlaid on the iUS images. **Table 2** summarizes the imaging characteristics of iUS at iUS-1, iUS-2 and iUS-3. We discuss three of these cases in detail below.

### Case 1

**Figure 1:**
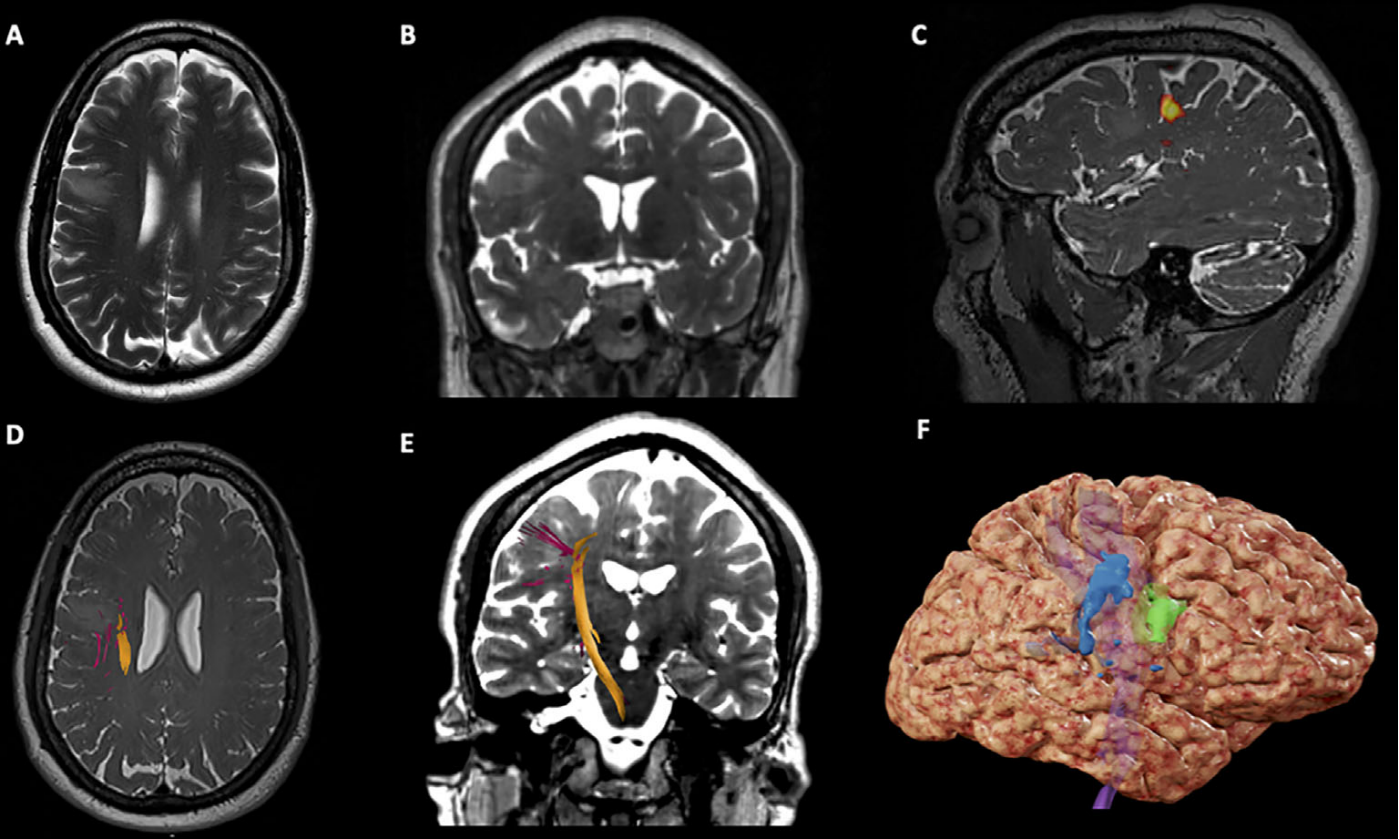
Case 1 Surgical Plan. **(A)** Axial T2-weighted imaging showing an hypointensity in the right middle frontal gyrus, anterior to the precentral gyrus; **(B)** Coronal T2-weighted imaging showing an hypointensity in the right middle frontal gyrus; **(C)** Sagittal T2*-weighted imaging showing BOLD activation for lip purse test (yellow) on the lateral aspect of the right precentral gyrus; **(D)** Axial T2*-weighted imaging showing the right Corticospinal Tract (CST) on the deep and posterior margins of the tumor; **(E)** Coronal T2-weighted imaging showing the right CST descending from the precentral gyrus in the posterior aspect of the tumor; **(F)** 3D brain reconstruction showing the BOLD activation for lip purse segmented (in blue), the segmented tumor (green) and the CST deep (in purple).

A 61-year-old right-handed man presented with new onset focal seizures, manifesting as dysarthria, and left-sided facial twitching. Brain MRI revealed a non-enhancing mass in the right frontal lobe anterior to the precentral gyrus (**Figure 1**). The lip pursing task from the preoperative fMRI showed BOLD activations in the pre- and postcentral gyri, 1cm posterior to the T2-hyperintense lesion (**Figure 1C**). The activations corresponding to the left hand were superior to the activations for lip pursing along the pre- and postcentral gyri, distant from the lesion. Language activations indicated left lateralized language function. White matter tractography seeded from motor fMRI BOLD activation areas was used to generate the right corticospinal tract (**Figures 1D, E**).

**Figure 2:**
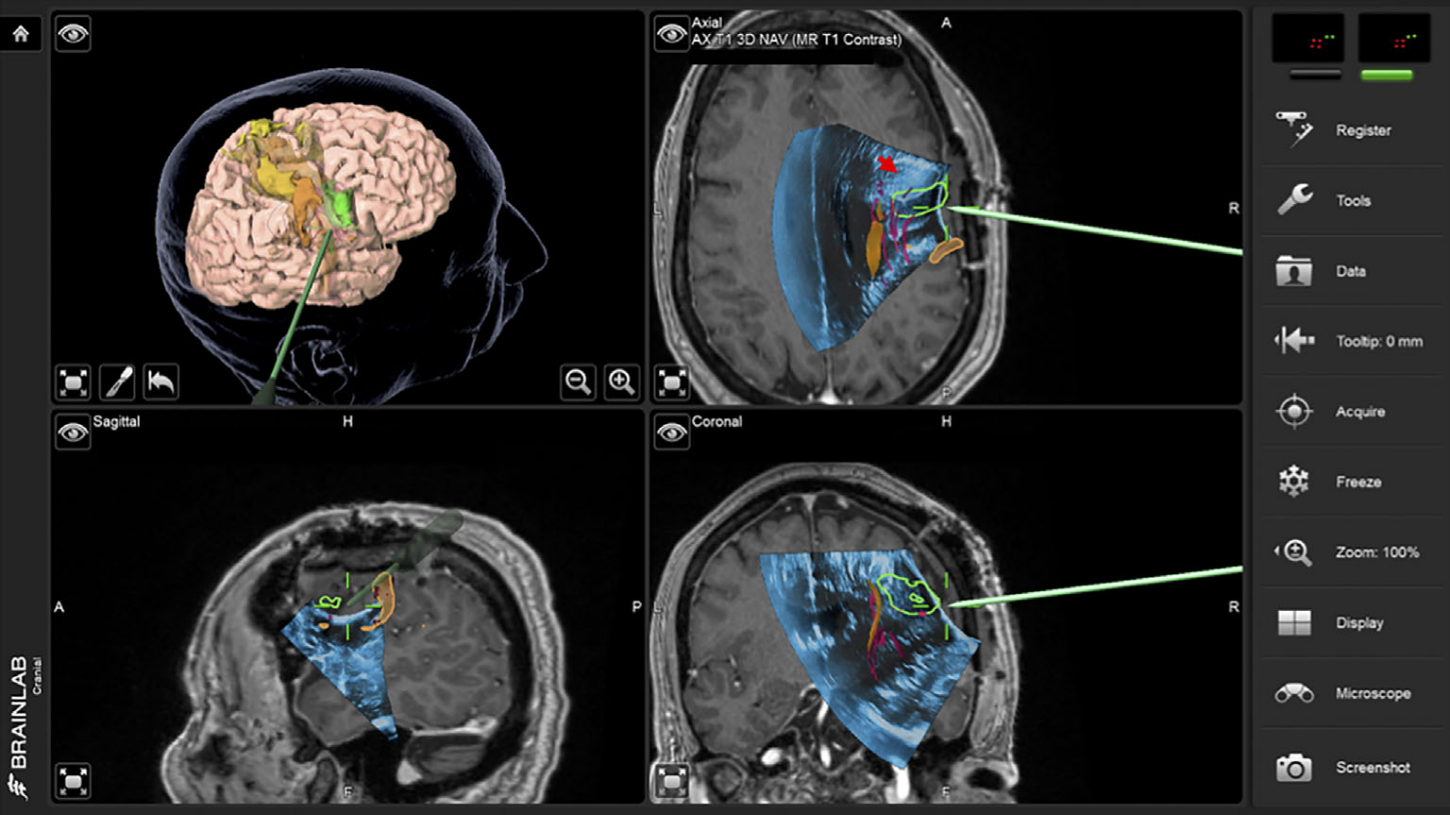
Case 1 intraoperative screen capture with the 3D surgical plan reconstruction with fMRI BOLD activation segmented in orange, tumor in green and Corticospinal Tract in yellow (the upper left panel), and the three orthogonal planes with 3D iUS-1 overlaid on preoperative MP2Rage. Notice the mismatch between the segmented tumor on MRI (green segmentation) and observed tumor on iUS (red arrowhead), placing the tumor site over the sulcus between the gyri making it difficult to know which gyrus was the tumor actually located.

**Figure 3:**
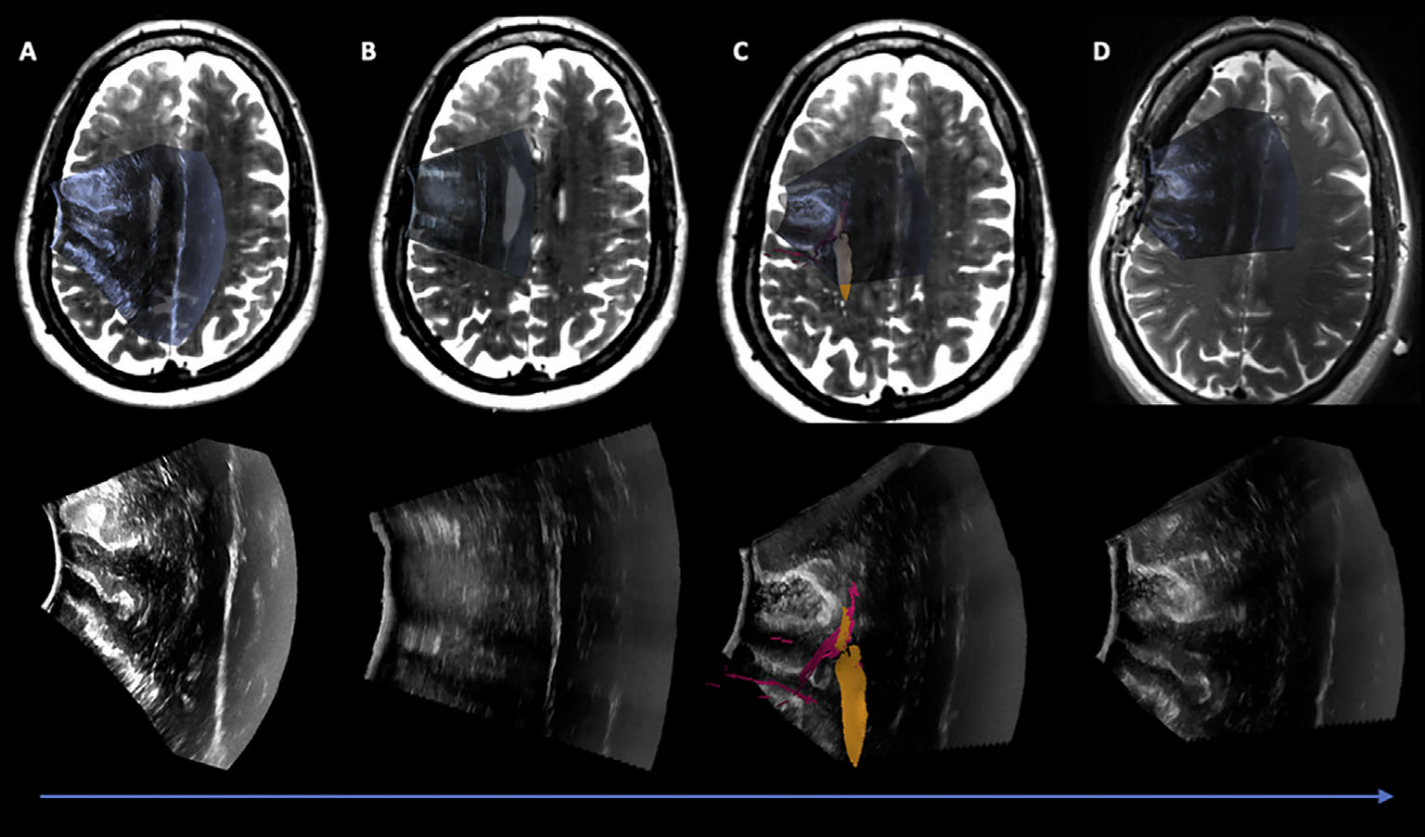
Case 1 Post hoc manually aligned preoperative axial T2-weighted imaging with **(A)** iUS-1, **(B)** iUS-2, **(C)** iUS-3 with right Corticospinal Tract (yellow) and Frontal Aslant Tract (red), and **(D)** iMRI axial T2-weighted imaging manually aligned with iUS-3. On D it is possible to observe a gross total resection on iUS and confirmed by iMRI.

A right frontotemporal craniotomy with transcranial and direct cortical motor mapping was performed. After the craniotomy, an extradural ultrasound (iUS-1) confirmed a homogeneously hyperechoic mass, expanding the gyrus anterior to the prefrontal gyrus (**Figures 2** and **3A**). Overlaying iUS-1 on the preoperative MRI showed a mismatch by one gyrus (approximately 1.5cm) between the segmented tumor on MRI and the tumor observed in the iUS (**Figure 2**). After the dural opening, iUS (iUS-2) was performed and the margins of the tumor were blurrier than iUS-1 (**Figure 3B**). The iUS (US-1 and US-2) in this case was used to precisely localize the area of the abnormality and the gyral anatomy. Cortical mapping was used directly over the planned resection sites and did not evoke any motor responses. Microsurgical resection proceeded with serial checks of the ultrasound to evaluate progress, especially as resection approached the posterior and medial margin of the lesion in close relation to the descending motor fibers (**Figure 3C**). Continuous subcortical motor mapping was also performed during the resection with a monopolar stimulating suction (Drytouch single-use Frazier Monopolar Stimulation Suction Probe, Neurovision Medical Products, Ventura CA). When a complete resection had been achieved per the surgeons estimate, a final iUS sweep (iUS-3) was acquired that suggested the absence of any residual tumor (**Figure 3D**) which was corroborated by the iMRI for confirmation of GTR. The patient had no postoperative neurological deficits and was discharged home after two days. Pathology revealed a glioblastoma multiforme.

### Case 2

**Figure 4:**
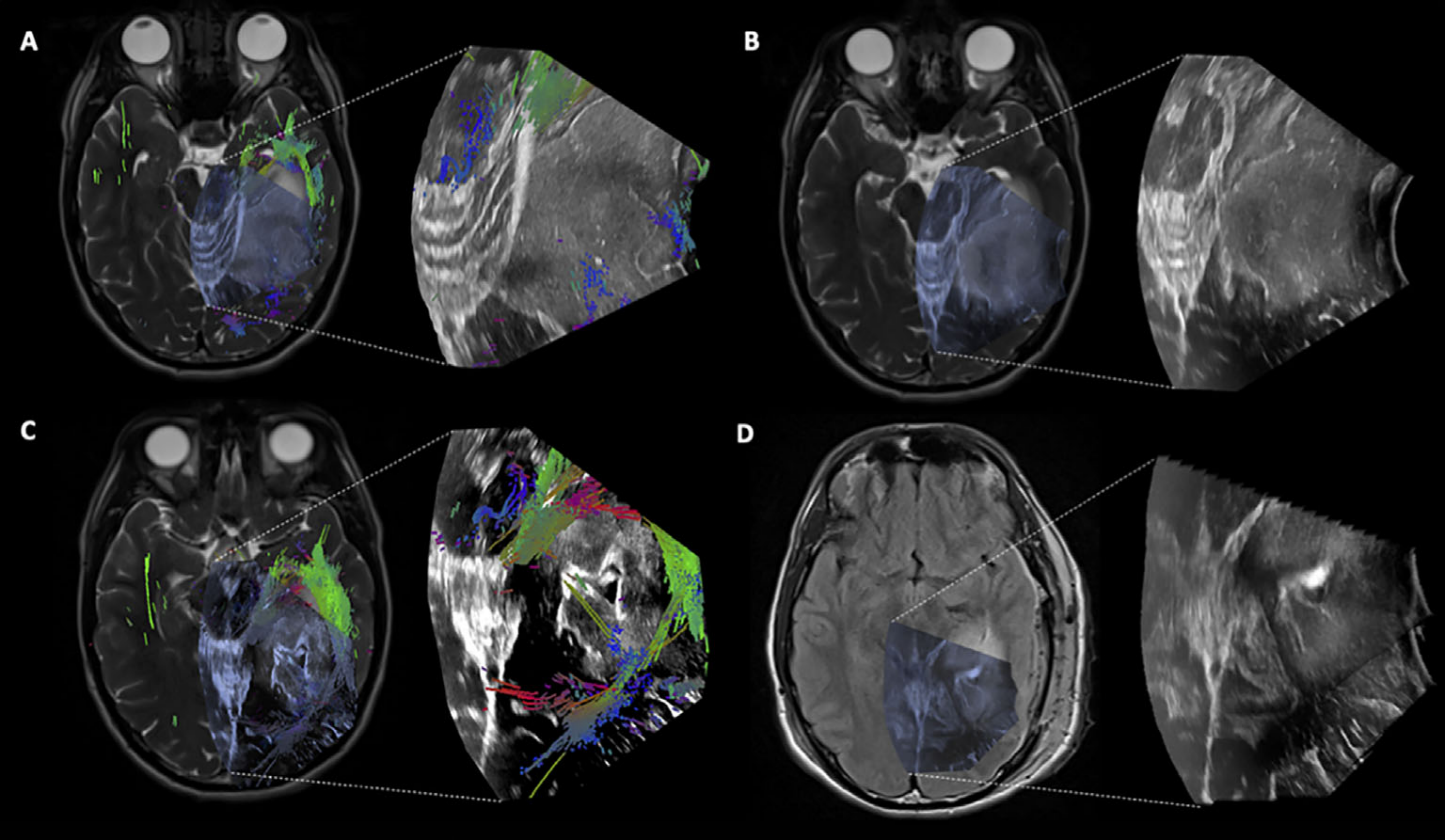
Case 2 Post hoc manually aligned preoperative axial T2-weighted imaging with **(A)** iUS-1, **(B)** iUS-2, **(C)** iUS-3 with multiple tracts around and inside the tumor, and **(D)** iMRI axial T2-FLAIR imaging manually aligned with iUS-3 without the overlaid tracts. Notice on C the presence of tracts running through the tumor. On D, it is possible to notice the presence of residual tumor in the posterolateral margin as well as posteriorly along the medial margin in the vicinity of the coursing tracts through the tumor.

A 28-year-old right-handed man presented with episodes of anxiety and fear lasting 20-30 seconds, with an initial diagnosis of post-traumatic stress disorder. Escalating frequency of these episodes prompted imaging evaluation, which revealed a large left non-enhancing mass in the mid- and posterior medial temporal lobe. Preoperative language fMRI showed BOLD activations in the putative receptive language areas of the left superior temporal gyrus, within 1cm of the T2-hyperintense lesion. Visual tasks were used to map the primary visual cortex using fMRI. The AF, IOFF/IFOF, ILF, FAT and Optic Radiations (OR) were created on the tractography software using fMRI BOLD activation areas as seed ROIs, consistent with their expected anatomical locations. An unexpected fiber bundle running through the middle of the posterior aspect of the lesion was suspected to be related to the visual pathway (**Figure 4**).

**Figure 5:**
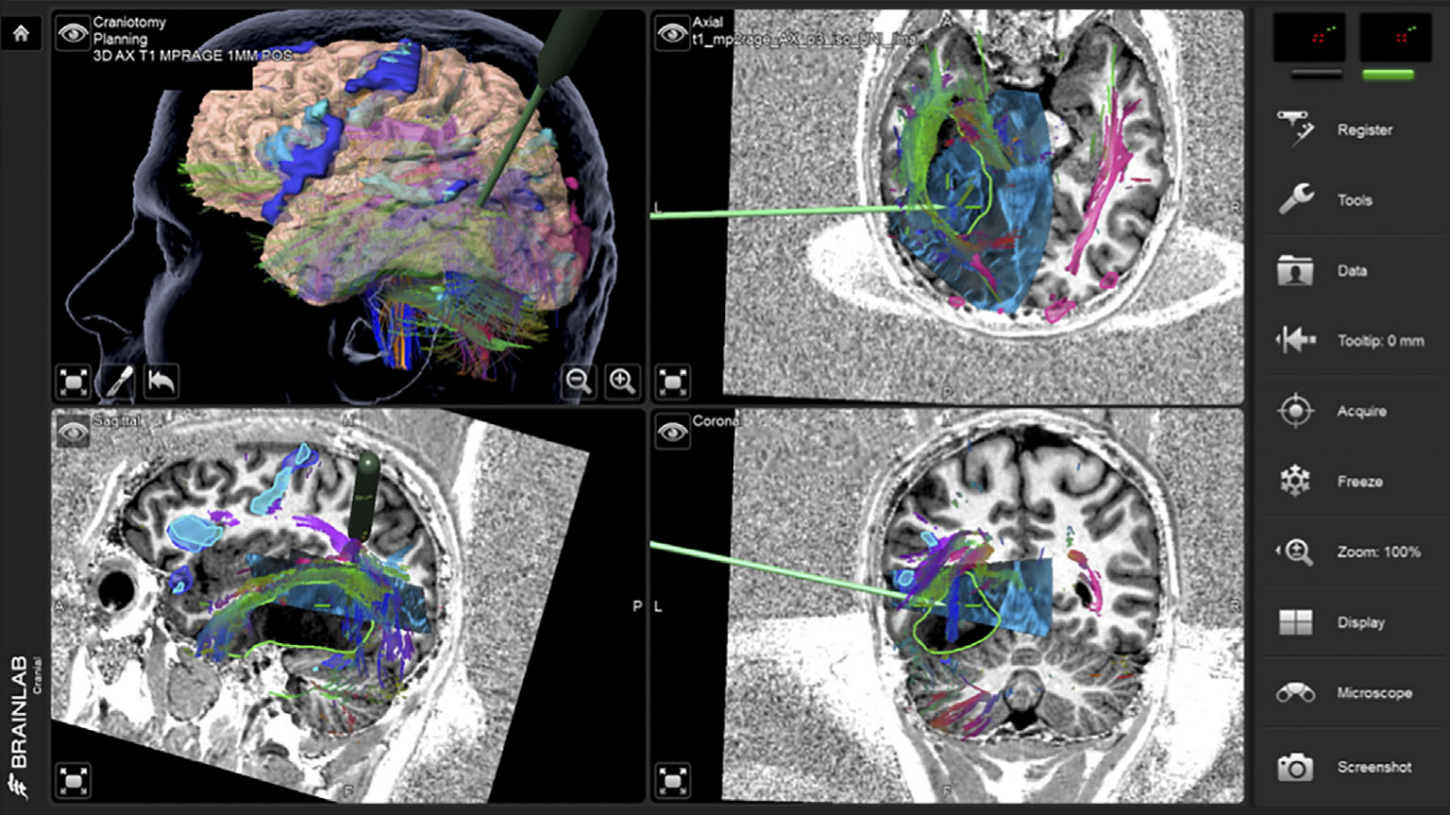
Case 2 intraoperative screen capture with the 3D surgical plan reconstruction with fMRI BOLD activation segmented in dark and light blue and tumor in green (upper left panel), and the three orthogonal planes with 3D iUS-3 overlaid on preoperative MP2Rage. Notice the navigation probe in close proximity to the tracts running through the tumor. In order to preserve these tracts, no further resection medially was performed.

A left temporal craniotomy was performed, with subsequent extradural ultrasound (iUS-1) which confirmed presence of a heterogeneous hyperechoic mass within the exposed area (**Figure 4A**). Relatively immobile reference landmarks, such as the tentorium, the brainstem, the cerebellum, and the cerebral aqueduct, were visualized as additional correlative landmarks. The dura was opened and an intradural ultrasound was performed to confirm the tumor location (**Figure 4B**). A linear pial incision, in the inferior temporal gyrus and parallel to the gyrus, was fashioned followed by dissection until the tumor was encountered. The tumor was extirpated centripetally, using multiple ultrasound sweeps to serially assess resection progress. At one point, the tracts within the tumor were localized using iUS and preserved (**Figures 4C** and **5**). When the initial resection goals were accomplished to the surgeons satisfaction, iUS-3 was performed, which suggested possible residual tumor at the posterolateral margin as well as posteriorly along the medial margin in the vicinity of the coursing tracts through the tumor (**Figure 4D**). iMRI confirmed the iUS findings (**Figure 4D**). The patient was re-draped, and the resection of the remaining tumor in the posterolateral aspect of the cavity was completed. The patient exhibited mild temporary word finding and reading difficulty early post-operatively. Three weeks after surgery, his preoperative anxiety attacks were nearly resolved and he only had minor word finding difficulties. On his 3-months follow-up, the patient was back to his baseline and seizure free. Pathology was compatible with a grade 3 *IDH1*-mutant anaplastic astrocytoma.

### Case 3

A 53-year-old woman presented with a partial motor seizure, with postictal transient right-sided hemiparesis. Brain MRI showed a non-enhancing deep-seated lesion in the left cingulate gyrus, extending to the mid-/posterior corpus callosum inferiorly and to the paracentral gyrus superiorly. Preoperative fMRI of hand clenching, finger tapping, and toe wiggling tasks showed BOLD activations in the anatomically expected M1 location and more anteriorly in the superior frontal gyrus. DTI tractography using fMRI BOLD activation areas as seed ROIs was used to generate the left CST and the left FAT. A 3D rendering of preoperative T1 post-contrast MRI reconstruction highlighted two prominent cortical veins entering the superior sagittal sinus in the planned craniotomy. Taken together, preoperative imaging suggested a narrow window for a safe operative corridor to the tumor.

**Figure 6:**
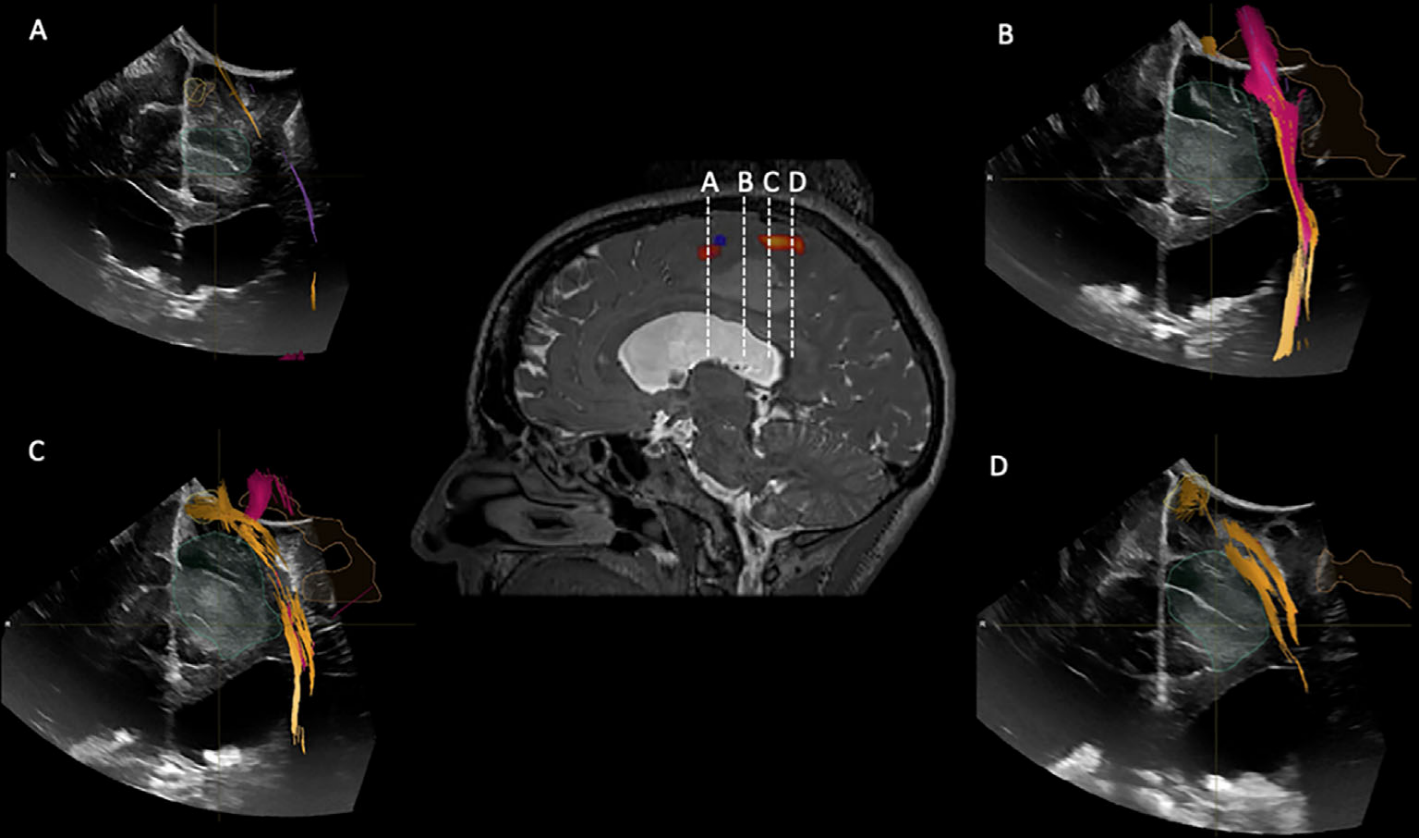
iUS1 in Case 3. Central panel shows a sagittal T2*-weighted imaging with fMRI BOLD activations for lower extremity (red) and upper extremity (blue). **(A)** Coronal iUS-1 showing the most anterior part of the tumor; **(B)** coronal iUS-1 showing the only possible surgical corridor that avoids the fMRI BOLD activations areas and Corticospinal Tract (CST); **(C)** Coronal iUS-1 overlaid with fMRI BOLD activation for lower extremity and CST superior to the tumor; **(D)** coronal iUS-1 with overlay of CST superior to the tumor.

**Figure 7:**
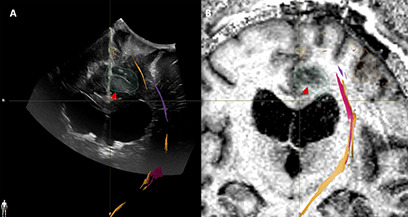
Case3. iUS3 **(A)** showing unexpected residual tumor (red arrowhead) under the arachnoid fold which was confirmed by iMRI **(B)**.

A left frontal craniotomy with transcranial and direct cortical motor evoked potentials as well as subcortical motor mapping was planned. The patient was positioned supine with head neutral and 30 neck flexion. Via a linear incision a craniotomy was performed to expose the superior sagittal sinus and coronal suture. iUS-1 confirmed adequate exposure of the tumor (**Figure 6**). A second iUS (iUS-2) was performed after the dura mater was opened to validate the continued accuracy of neuronavigation; there was a potential for a brain shift in the anteroposterior direction due to cerebrospinal fluid (CSF) drainage but none was observed. We attempted an interhemispheric dissection, which was limited by the bridging veins merging with the sinus along its length, prompting pursuit of a transcortical approach instead. The cortical entry point was defined between the two hand and foot activation areas connected by the corticospinal tract and the frontal aslant tract, using a combination of motor mapping and navigating the iUS-2 volume with the preoperative MRI (**Figure 6B**). The anterior-most and superficial part of the tumor was well differentiated from the surrounding brain, but the posterior and deeper portion was only partially distinguishable. Surgical resection was pursued posteriorly until subcortical stimulation of motor tract responses reached a threshold of 2.5 mA. A third iUS sweep (iUS-3) indicated some residual tumor in the anterior-most part of the cavity, cloaked by a sheath of arachnoid in the cingulate sulcus (**Figure 7A**). iMRI at this point confirmed residual tumor in both the anterior and posterior aspects of the surgical cavity (**Figure 7B**). Additional microscopic resection of the anterior residual tumor was achieved. In the early postoperative period, the patient exhibited right-sided hemiparesis, most prominent on lower extremity with preserved tone, with near-complete recovery by postoperative Day 10 barring minor gait apraxia, and a full recovery after 3 weeks consistent with a supplementary motor syndrome. Pathology was compatible with glioblastoma multiforme

## Discussion

### 2D and 3D iUS Compared to Preoperative MRI

In the present paper, the locations and margins of the tumors as shown by iUS were in agreement with preoperative MRI. This is consistent with findings in the literature of significant correlation between 2D US and preoperative MRI (28, 29). While there is additional literature on the ability of iUS to provide better tumor delineation than T1-weighted MRI iUS, (30; G. 31), we have not systematically studied this.

### iUS and Navigation

Navigated 3D iUS was able to provide real-time information regarding brain shift, possible problems with registration and to identify potential surgical entry points in all cases. Since we used iUS-1 and iUS-2 to validate neuronavigation on preoperative MRI, we were able to recognize rigid mismatches and avoid errors such as incision of the incorrect gyrus that could have happened if we had navigated solely on the preoperative MRI (Case 1). iUS allowed us to enhance neuronavigation by providing important information regarding the best window between two functional areas to proceed with a corticectomy (Case 3). A recent study of 210 glioma patients reported that serially acquired navigated iUS played an important role in assessing resection progress (32).

### iUS to Measure and Monitor Brain Shift

An important advantage of iUS is its ability to monitor brain shift over the course of surgical resection and to provide real-time anatomical information that reflects the current intraoperative state. In our cohort, we observed minimal brain shift between iUS-1 and iUS-2, but as the surgical resection progressed, the mismatch between the preoperative MRI on the navigation system and the iUS became evident, rendering neuronavigation suboptimal for clinical decision-making. Therefore, the only true, real-time image-guidance available to us was from iUS. While we have not employed any non-rigid registration methods in this study, we and others have developed several that have continued to move the field forward for registration of MRI-MRI and MRI-iUS volumes (6, 27, 33,37).

### Concordance Between iUS and iMRI

Our study showed a concordance rate of 100% between iUS and iMRI findings in predicting the EOR in patients where good contact between the US probe and the brain surface was possible; on iUS-3 four patients showed no residual tumor and fifteen patients showed residual tumor, all confirmed by iMRI. These results are similar to the earliest study that revealed a 100% concordance rate between the two imaging modalities (28). (38) showed in a study of 20 GBM surgeries that tumor detection sensitivity using a navigated linear array ultrasound transducer is significantly higher (78% vs. 24%) compared to using a curved array transducer, while specificity is reduced (from 58% vs 96%). A more recent study in both adult and pediatric patients with brain tumors using iUS showed 81% concordance, with 19% false-negative results (39). The main hurdle in using iUS to establish the EOR is the difficulty by the end of the surgery to assess if a hyperechoic area is truly residual tumor, since the enhancement artifact caused by differences in attenuation of the resection cavity fluid and the surrounding brain is a significant surgically induced ultrasound artifact (40). Although we noticed this artifact in some of our cases, we were able to confirm the iUS findings using iMRI because all the cases were performed in the AMIGO suite. A potential solution to this problem is a coupling fluid that attenuates ultrasound energy like the normal brain and reduces enhancement artifacts, which has been studied though is not yet commercially available (40).

### Limitation of iUS Imaging

In patients with large resection cavities, either new or previous, contact between iUS probe and the brain surface can be difficult or impossible to achieve, particularly in cases where the head position does not favor retention of fluid in the cavity. In such cases we were unable to obtain 2D and hence 3D iUS.

### Challenges in Creating 3D iUS Volume

Although 3D iUS provides us with a powerful surgical tool, creating these volumes poses some challenges. It is difficult to maintain a uniform speed of probe translation while collecting 2D images for a volume. This task becomes more difficult later in the surgery when the resection cavity is filled with fluid and subtle variations in the steadiness of the transducer leads to artifacts in the resultant 3D volume.

## Conclusion

The impact of iUS on neurosurgical practice continues to evolve in the face of improved transducers, neuronavigation systems, and surgical technique. This review of the recent experience at our institution illustrates the practical benefits of iUS in relation to iMRI, and the challenges encountered in a range of tumor resection cases.

## Data Availability Statement

The raw data supporting the conclusions of this article will be made available by the authors, without undue reservation.

## Ethics Statement

The studies involving human participants were reviewed and approved by Mass General Brigham Institutional Review Board. The patients/participants provided their written informed consent to participate in this study. Written informed consent was obtained from the individual(s) for the publication of any potentially identifiable images or data included in this article.

## Author Contributions

Data collection: DB, PJ. Data interpretation: DB, PJ, AG, WB, TK, SF. Manuscript: DB, PJ, AG, WB, TK, SF, SP. Manuscript editing: DB, PJ, AG, WB, TK, SF, YT, SP, WW. Bibliography editor: DB, NJ, TK. Images editor: DB, PJ. All authors contributed to the article and approved the submitted version.

## Conflict of Interest

The authors declare that the research was conducted in the absence of any commercial or financial relationships that could be construed as a potential conflict of interest.
